# Statistical Study of Whistler‐Mode Waves and Expected Pitch Angle Diffusion Rates During Dispersionless Electron Injections

**DOI:** 10.1029/2021GL094085

**Published:** 2021-09-03

**Authors:** R. Ghaffari, C. M. Cully, C. Gabrielse

**Affiliations:** ^1^ Department of Physics and Astronomy University of Calgary Calgary AL Canada; ^2^ The Aerospace Corporation El Segundo CA USA

## Abstract

Energetic electron injections can generate or amplify electromagnetic waves such as whistler‐mode waves. These waves can resonantly interact with available particles to affect their equatorial pitch angle. This process can be considered as a diffusion that scatters particles into the loss cone. This study investigates whistler‐mode wave generation in conjunction with electron injections using in situ wave measurements by the Time History of Events and Macroscale Interactions during Substorms mission during 2011–2020. We characterize the whistler‐mode wave behavior associated with 733 selected dispersionless electron injections and dipolarizing flux bundles (DFBs). We observe intense wave activity and strong diffusion associated with only the top 5% and 10% of the selected injection events, respectively. We also study the wave activity when there is a sharp rise in the northward component of the magnetic field around the injection time (DFBs). In this case, the generated wave powers increase, and the power change is at least two times greater than non‐DFB injections.

## Key Points

- Analyzing 733 dispersionless electron injections, only 5% show an increase in whistler‐mode wave power by more than an order of magnitude
- Roughly, 10% of the analyzed injections show a measurable increase in diffusion rate
- Greater increases in wave power are observed when the injection is coincident with magnetic field dipolarization

## 1 Introduction

Wave‐particle interactions play an important role in the dynamics of the inner magnetosphere. They can cause major acceleration or loss of the trapped population and affect the whole geospace system dynamics, especially during high geomagnetic activity periods (e.g., Reeves et al., 2003). When magnetospheric waves resonantly interact with the trapped particles, the particles' pitch angle is affected, and they may enter the loss cone through a diffusion process and precipitate into the atmosphere. In particular, when the perpendicular flux exceeds the parallel flux, we can expect generation and/or amplification of whistler‐mode waves (Baker et al., 1981) through different mechanisms (e.g., Gao et al., 2014a, 2016) and possible resonant interactions with the particles and consequent precipitation into the atmosphere.

There is a huge body of work on pitch angle scattering of trapped particles in the magnetosphere through resonant interaction with electromagnetic waves (e.g., Albert, 2005; Kennel & Engelmann, 1966; Kennel & Petschek, 1966; Lyons, 1974a, 1974b; Lyons et al., 1971; Schulz & Lanzerotti, 1974; Summers et al., 1998). Generally, the trapped population is considered in cyclotron resonance with the electromagnetic waves, affecting the particle's pitch angle. Consequently, wave alteration of the pitch angle may alter the first adiabatic invariant of the motion and lead to particle scattering into the loss cone and precipitation. This diffusion is studied by solving a Fokker‐Planck equation (Roberts, 1969) and calculating diffusion coefficients. In weak plasma turbulence, wave‐particle interactions can be described by the quasi‐linear diffusion theory, with pitch angle diffusion resulting from cyclotron resonant wave‐particle interactions (Summers et al., 2005). Horne and Thorne (1998) indicate that in low density regions outside the plasmasphere, whistler‐mode waves can resonate with electrons over the energy range around 100 keV to 1 MeV. Summers et al. (1998) advanced the theory of wave‐particle cyclotron resonant diffusion and constructed diffusion curves for electron cyclotron resonance with field‐aligned R‐mode and L‐mode electromagnetic waves under relativistic conditions. Summers (2005) derived a relativistic formula for the pitch angle diffusion coefficient for cyclotron resonant interaction with electromagnetic waves including whistler‐mode waves.

Statistics of the electromagnetic waves in the Earth's magnetosphere, including whistler‐mode waves and diffusion rates, have been reported in numerous studies (e.g., Gao et al., 2014b; Horne et al., 2013; Li et al., 2007, 2009, 2011, 2013; Ma et al., 2016; Meredith et al., 2014; Ni et al., 2014). For instance, Li et al. (2011) studied the distribution of the chorus waves from Time History of Events and Macroscale Interactions during Substorms (THEMIS) wave spectra data in different Magnetic Local Time (MLT) sectors and different geomagnetic activity levels. They showed that strong waves are mainly expected in L‐shells lower than 8, and the wave amplitude increases with increasing Auroral Electrojet index. Cully et al. (2008) studied whistler‐mode waves in the inner magnetosphere observed by the THEMIS. They showed a high‐amplitude tail in the wave power probability distribution caused by bursty waves across extended regions in space. As discussed in Cattell et al. (2008) and Santolík et al. (2003), the trapped particles' acceleration can result from the small number of interaction with extremely intense waves rather than frequent interactions with weak waves.

Energetic particle injection is one type event that can cause anisotropic distributions and result in the generation and amplification of various waves, including whistler‐mode waves (e.g., Meredith et al., 2001; Rodger et al., 2016). Injections are usually associated with substorms in the magnetotail (e.g., Baker et al., 1982, 1996; McIlwain, 1972), and they are identified as sudden increases in particle flux over a broad energy range. When there is not any time shift between flux enhancements at various energies, it is referred to as a “dispersionless injection.” If the spacecraft encounters the particle injection farther from its source, it is called a “dispersed injection,” and there is a delay in flux enhancement between particles with different energies due to energy dependent gradient and curvature drifts (e.g., Zaharia et al., 2000).

Dipolarizing flux bundles (DFBs), which have a strong correlation with energetic particle injections (Moore et al., 1981; Runov et al., 2009, 2011), are also important in the generation of electromagnetic waves (e.g., Sergeev et al., 2009; M. Zhou et al., 2009). DFBs are regions with a sharp increase in the northward component led by the dipolarization front in the Earth's magnetosphere. These are localized (few $R_{E}$) areas of magnetic dipolarization which have been observed in the magnetotail plasma sheet extending to at least 30 $R_{E}$ (Liu et al., 2013). Plasma sheet electrons are accelerated at the strong magnetic and electric fields at the DFB's edge, which result in hotter and more energetic electrons than the ambient plasma sheet (Birn et al., 2014; Fu et al., 2011; Lu et al., 2016). Like energetic particle injections, earthward transport of electrons during DFBs may lead to temperature anisotropy and play a significant role in generating a variety of electromagnetic waves, including whistler‐mode waves (Zhang et al., 2019).

Previous studies, including global distribution of waves (as mentioned above), are relevant to long‐term averaged conditions. However, they are not necessarily indicative of the state of the system at any given time. Since there is considerable precipitation immediately following injection, it is reasonable to ask what the typical wave power is immediately following the injection. In this study, we investigate the incident frequency and amplitude distribution of the whistler‐mode waves in conjunction with energetic electron injections to quantitatively understand the role of wave‐particle interactions in the magnetospheric dynamics during these specific periods. Initially, we compile a list of 733 dispersionless electron injections following the criteria of Gabrielse et al. (2014), using THEMIS in situ measurements. In Section 3, we present a case study of an injection event and calculate its diffusion rate to compare with the strong diffusion rate. In Section 4, we perform a superposed epoch analysis on all of the selected electron injection events to study their typical behavior and find out how frequently we observe enhanced wave activity in conjunction with the events. Later, we calculate the diffusion coefficient during the injection events to compare with the strong diffusion limit.

## 2 Data and Methodology

The THEMIS mission operates five satellites in highly elliptical orbits. The satellites have been equipped with identical instruments for observing the in situ particles and fields (Angelopoulos, 2009). In this study, we rely on THEMIS‐D fast survey data through 2011–2020 obtained while the spacecraft was in the plasma sheet (β>0.5). We use energy flux from the Solid State Telescope (SST) which measures the plasma flux at energies from 30 keV to 4 MeV (Angelopoulos, 2009). We smoothed the electron energy flux by 4 min (equivalent to 80 data points). The DC magnetic field data is measured by the fluxgate magnetometer (FGM) instrument (Angelopoulos, 2009; Auster et al., 2008). Magnetic field waveform is measured by the Search Coil Magnetometer (SCM; Le Contel et al., 2009; Roux et al., 2008), with wave power computed by the Digital Fields Board (DFB; Cully et al., 2009) in 16, 32, or 64 frequency bins covering the bandwidth from 0.1 Hz to 4 kHz along three spacecraft axes (*X*, *Y*, and *Z*). Calculated moments including electron and ion density are provided by the electrostatic analyzer (ESA) instrument (McFadden et al., 2008). We also use plasma density inferred from the spacecraft potential following Nishimura et al. (2013) using the method proposed by Mozer (1973) and Pedersen et al. (1998).

To quantitatively define dispersionless electron injections and avoid selecting random particle flux fluctuations as electron injections, we followed the selection criteria defined by Gabrielse et al. (2014). The selection criteria for injections are as follows: For three consecutive energy channels, (a) the energy flux (*j*) rate of increase reaches (Δ(j)/j)/Δ(t)>25%/ minute. (b) The temporal separation of the lowest three consecutive energy channels at the injection onset (*t*_0_) is less than 1 min to be considered as dispersionless injection. (c) The average energy flux 10 min after *t*_0_ is at least two times greater than the 10‐min average beforehand. (d) The energy flux remains elevated for at least 5 min (e) β>0.5 for 15 consecutive minutes surrounding *t*_0_ to distinguish an electron injection from any other fluctuation when the satellite crosses the plasma sheet to the lobe region. We also disqualified the electron injection if the energy flux at *t*_0_ for the first three SST energy channels (∼30−50 keV) was below $5\times 10^{3}$ keV (str.cm^2^.s.keV). We did not select any new onset within the 10‐min following an injection.

## 3 Case Study

In this section, we present two case study injection events and their associated wave power in ±1 hour around the detected onset. These two examples are two typical events that we observed in our selected events list and we discuss them in more detail. We calculate the expected diffusion coefficient for one of the events and compare it to the strong diffusion rate.

### 3.1 Observations

**Figure 1:**
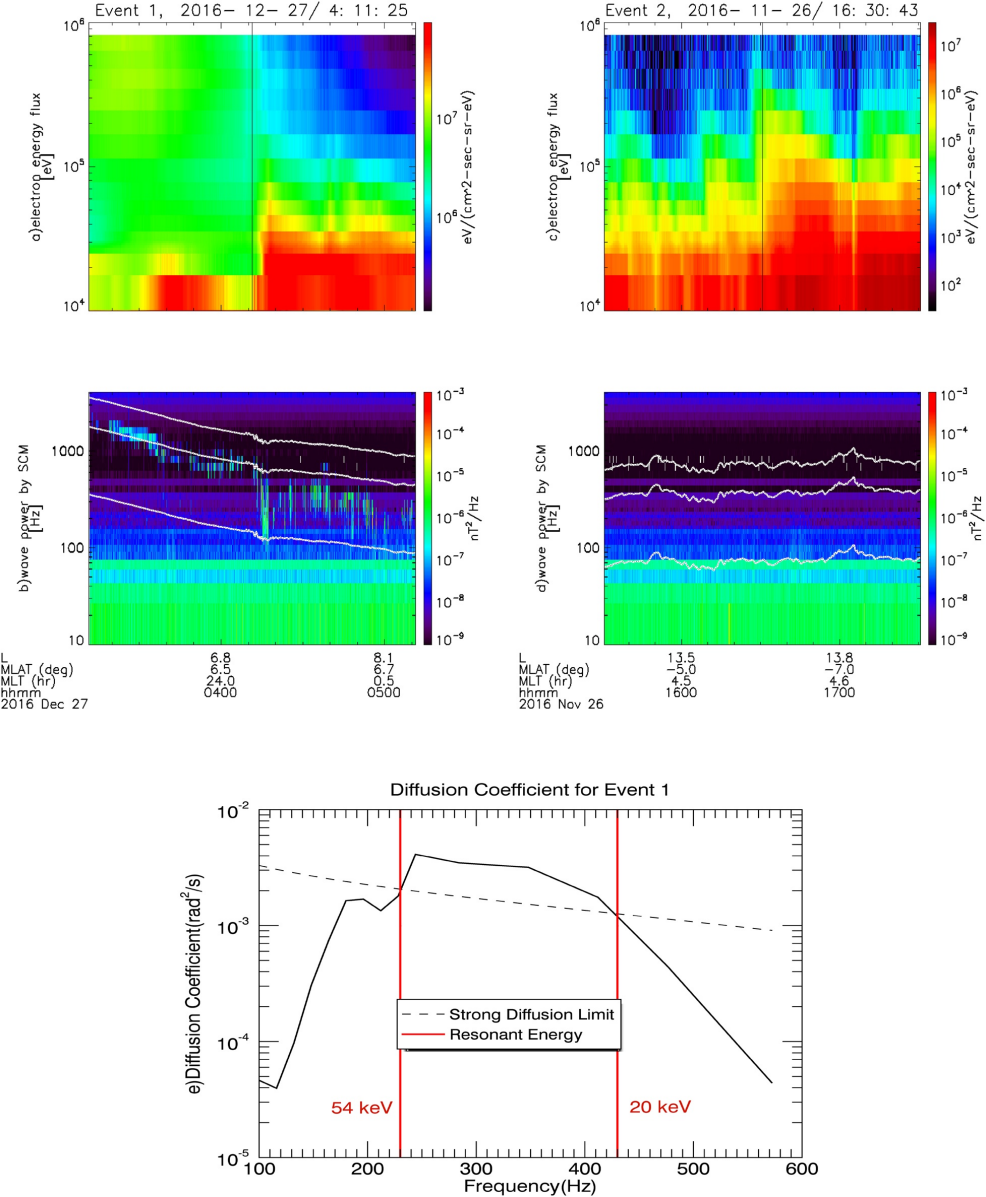
Two examples of selected injections, showing electron energy flux measurements (panels (a) and (c)) where the vertical black lines indicate the injection time and associated SCM measurements of the wave activity (panels (b) and (d)). The white curves are $f_{ce}$ (gyrofrequency line), $0.5f_{ce}$ and $0.1f_{ce}$. Event 1 shows strong electromagnetic wave generation, whereas there is no associated wave generation for Event 2. Panel (e): The solid black curve is the calculated diffusion coefficient for the case study Event 1. The dashed curve is the expected strong diffusion rate for this event. The vertical red lines mark the edges of the resonant frequency band, and are labeled with the parallel energies corresponding to those frequencies for this event. Particles between roughly 20 and 54 keV will be strongly scattered.

Figure 1 shows two examples of events selected as injections (Event 1 and Event 2). Panels (a) and (c) show the electron flux measured for electrons above 10 keV from combined measurements of ESA and SST for both events; the sudden enhancement in the flux indicates the electron injections. Panels (b) and (d) show the wave power measured by SCM over different frequency bands and the white curves are $f_{ce}$ (gyrofrequency line), $0.5f_{ce}$ and $0.1f_{ce}$. The magnetic field power spectrum is onboard Fast Fourier Transformed data (FFT). In panel (b), we can see electromagnetic wave generation associated with the observed injection and enhanced power after the onset. We interpret these strong electromagnetic waves as whistler‐mode waves based on their frequency range and electromagnetic nature, although the polarization cannot be determined from the FFT data. Amplification and generation of electromagnetic waves is not always observed following injection, and we found many injections with no wave signature such as Event 2 in Figure 1.

We also investigated the northward Geocentric Solar Magnetospheric component of the magnetic field to determine if these selected events are among DFBs as a subclass of injections or not. We checked the *B*_*z*_ component within 2 min before to 15 min after the injection time to determine whether they satisfy the most important characteristic of a DFB event as $\partial B_{z}/\partial t>0.5$ nT/s (Zhang et al., 2019). Neither of these case study events showed a sharp increase in the *B*_*z*_ as we expect for DFBs.

### 3.2 Diffusion Coefficient

We can characterize the diffusion and wave power based on in situ measurements. In this section, we estimate the diffusion coefficient for the Event 1 in Figure 1. We calculate diffusion rates for each event following Summers (2005), using measured wave power and plasma density.

Considering the gyro‐resonance condition as $\omega -k_{∥}v_{∥}=\Omega /\gamma$, and using Summers (2005) Equation 17 to calculate the quasi‐linear diffusion coefficient for field‐aligned electromagnetic waves such as whistler‐mode waves, we estimate the pitch angle diffusion coefficient as a function of wave frequency. Here, ω is the resonance wave frequency, k is the wave number, v is the particle velocity, Ω is the particle gyrofrequency and γ is the Lorentz factor. We obtained wave power from in situ measurements along the satellite trajectory. Because wave power measurements are only available at the satellite, we consider only the local scattering rate and not a bounce‐averaged scattering rate. As whistler‐mode wave growth rate maximizes for parallel propagation (Horne et al., 2003) and most whistler‐mode waves observed by THEMIS in Li et al. (2011) statistical study are parallel propagating, we assumed the observed wave distribution is parallel propagating with zero normal angle. The assumption of parallel propagation tends to slightly overestimate the diffusion rates (Shprits et al., 2006). For simplicity, we take the wave power as the averaged power in an hour after the injection time to calculate the diffusion coefficient.

We also estimated the strong diffusion rate following Kennel (1969) as $D_{SD}=\frac{2\alpha_{LC}^{2}}{\tau_{B}}$ where $D_{SD}\;$ is the strong diffusion rate, $\alpha_{LC}\;$ is the atmospheric loss cone, and $\tau_{B}$ is the bounce period for particles. To calculate $\alpha_{LC}$ $\left(\sin\alpha_{LC}=\sqrt{B_{eq}/B_{A}}\right)$, we traced the field lines to the equator, using T89 with $K_{p}=3$ (Tsyganenko, 1989) as the magnetic field model to estimate magnetic field strength at the equator ($B_{eq}$). We assumed the magnetic field strength $B_{A}$ at the ionospheric footprint is $5\times 10^{4}$ nT. We estimated $\tau_{B}$ based on the expected resonant energies and the magnetic field line length that particles travel in each bouncing motion (C. Zhou et al., 2013).

The solid black curve in Figure 1e shows the calculated diffusion coefficient for the case study Event 1. It clearly indicates enhanced diffusion rate over a range of frequencies as a result of waves generated during the injection. The dashed curve in this figure is the expected strong diffusion rate for this event. The observed scattering rate exceeds the strong diffusion limit in this case. Vertical red lines are the expected parallel resonant energy range calculated using electron density from ESA. In this event we expect that ∼20–54 keV electrons effectively interact with the observed whistler‐mode waves and are strongly scattered.

## 4 Superposed Epoch Analysis

Following the event selection criteria discussed in Section 2, we found 733 dispersionless electron injections on THEMIS‐D between 2011 and 2020 when the spacecraft was in the magnetotail. The events' distribution in MLT and L is shown in the supporting information. Starting from the list of selected electron injections, we extracted the magnetic field power spectrum over 32 frequency bins measured by SCM in THEMIS‐D in both *Y* and *Z* directions for each event and considered the sum of these two powers in this analysis as the wave power. We used FFT power spectra of the magnetic field in fast survey mode (the FFF data product) in this work. Then, we calculated the integrated wave power between 0.1 and 0.5 electron gyrofrequency (as typical lower band chorus) for each event. Finally, we calculated temporal averages and smoothed the data to use 1 min averaged power for ±60 minutes around the injection time.

### 4.1 Electron Injections

**Figure 2:**
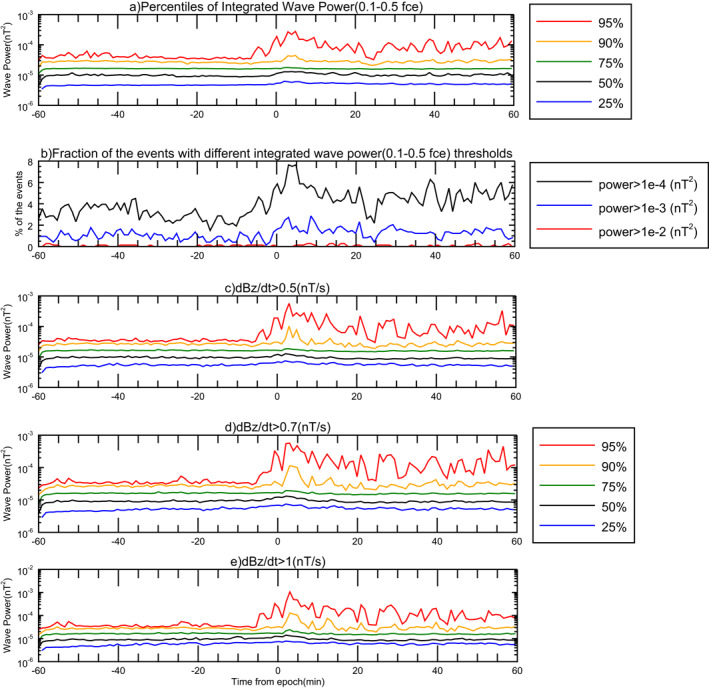
Superposed epoch study of different parameters: (a) the integrated wave power (0.1–0.5 electron gyrofrequency bands) during 733 selected injection events, (b) the statistics of the integrated waves power magnitude (0.1–0.5 electron gyrofrequency) over time during 733 selected injection events, (c) the integrated wave power (0.1–0.5 electron gyrofrequency bands) during 331 selected events with $\partial B_{z}/\partial t>0.5$ nT/s, (d) The integrated wave power (0.1–0.5 electron gyrofrequency bands) during 257 selected events with $\partial B_{z}/\partial t>0.7$ nT/s, (e) The integrated wave power (0.1–0.5 electron gyrofrequency bands) during 180 selected events with $\partial B_{z}/\partial t>1$ nT/s.

Figure 2a presents several percentiles ranging from 25% to 95% of the integrated wave power in lower band chorus for ±1 hour around the injection time. For almost all of the curves we observe a change in the wave power at the epoch time. The observed integrated wave power curves mostly do not show a large enhancement, with the wave power up to ∼2 times greater after the epoch time in comparison with beforehand. The 95th percentile (red curve) varies much more and shows around an order of magnitude enhancement in the wave power. This figure also represents a heavy tail wave power distribution. We observe much higher generated wave powers in higher percentile. This is consistent with previous THEMIS observations (Cully et al., 2008).

We also looked at the wave power distribution as a function of time during our selected events. Figure 2b shows statistics of the integrated wave power magnitude (between 0.1−0.5 electron gyrofrequency) over time. It shows the percent of the events with powers greater than three selected thresholds of $10^{-2},\;10^{-3}$ and $10^{-4}$ nT^2^ at any moment. We observe that integrated powers greater than $10^{-4}$ nT^2^ are very rare. However, just as shown in panel (a), the tail of the occurrence distribution is enhanced after the injection.

### 4.2 Dipolarizing Flux Bundles

We also investigated magnetic field changes around the selected injections to determine if any DFBs are associated with these injections. We categorized the events into three different groups with $\partial B_{z}/\partial t>0.5,0.7,1$ nT/s for −2, +15 min around the injection time to study possible impacts. We used 8‐s cadence magnetic field data in this part.

Figures 2c–2e present several percentiles of the integrated wave power in lower band chorus for ±1 hour around the injection time for some specific thresholds of *B*_*z*_. Panel (c) shows the integrated wave power for the case of $\partial B_{z}/\partial t>0.5$ nT/s. We obtained 331 events from the list that satisfy this criteria. The 95th percentile (red curve) shows an enhancement of ∼19 times greater power after the epoch time compared to beforehand in this case. This value is roughly double the power enhancement of the red curve in Figure 2a, where we did not consider any criteria for *B*_*z*_ component during the event. Similarly, panels (d) and (e) show different percentiles of the wave power for the cases of $\partial B_{z}/\partial t>0.7$ and $\partial B_{z}/\partial t>1$ nT/s, where we have 257 and 180 events, respectively. The 95th percentiles (red curves) here show an enhancement of ∼20 and ∼35 times greater power after the epoch time compared to beforehand respectively. We conclude that the wave power enhancement is greater in events with a sharp rise in the northward component of the magnetic field (DFBs). DFBs can be considered a particular class of injections, and with strong changes in the magnetic field inside DFBs, we expect a more dramatic change in wave power. Interestingly, for both DFBs and all other injections, the significant change in the wave power happens only for the 95th percentile (red curves in Figures 2a, 2c–2e).

We classified the events into two groups based on their L‐shell, L>10 and L<10, and performed the same analysis (see Supporting Information). For both groups, the visible change in the wave power happens only for the top two percentile ranges (90–95th).

### 4.3 Diffusion Coefficient

**Figure 3:**
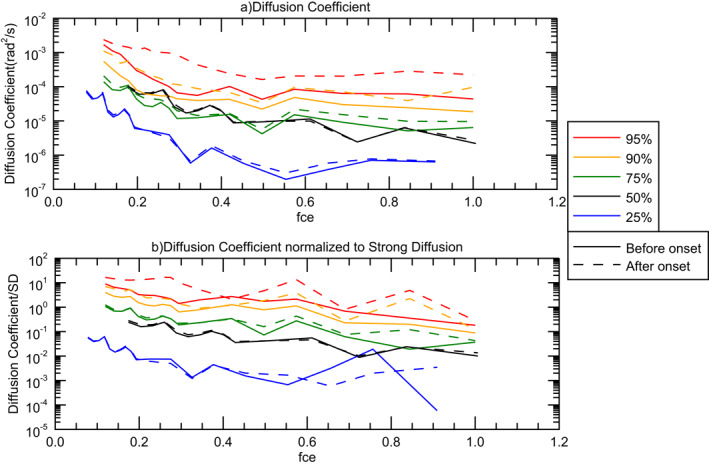
Different percentiles (ranging from 25% to 95%) of the calculated diffusion coefficient (panel (a)) and diffusion coefficients normalized to strong diffusion rate (panel (b)) for before and after the injection onset (solid and dashed curves, respectively). *X*‐axis is the frequency normalized to the gyrofrequency‐$f_{ce}$.

Finally, we calculated diffusion coefficients for all selected events as described above for the case study (Section 3.2). Due to the lack of observation of the upper hybrid line in the THEMIS plasma data, we are not able to estimate the electron density precisely. In this part, we used calculated electron density from spacecraft potential measurements. Solid curves in Figure 3a present the diffusion coefficient percentiles (25%–95%) before the injection onset and dashed curves present the percentiles after the injection. Comparing solid and dashed curves indicates there is almost no change observed in the diffusion rates for at least ∼75% of the events. Panel (b) is the diffusion coefficient normalized to the strong diffusion rate for each event. Same as panel (a), solid curves are for before the injection onset and dashed curves are for after the injections. We observe that the top 25% of events are very close to or above the strong diffusion limit; meanwhile the normalized diffusion rate show an enhancement only for top 10% of the events. Most of the events (at least 75%—green curve) do not show a change before and after the injection.

We conclude that based on the observed wave activity in conjunction with electron injections, only a small fraction of the events (top 10%) are associated with enhanced diffusion rates. It is worth emphasizing that here we assumed all the measured wave power is related to whistler‐mode waves and reported the expected scattering due to these presumed whistler‐mode waves. We do not infer anything about precipitation due to other wave modes or mechanisms.

It is important to note that the diffusion coefficient and strong diffusion limit are sensitive to the electron density measurements which introduce some uncertainty in strong scattering rates reported in this figure. We also calculated diffusion rates by considering electron density from ESA plasma instrument measurements rather than spacecraft potential estimation (as shown in Figure 3). In this case, still the top 25% of the events are close to or above the strong scattering limit, but the 90–95th percentile curves are slightly shifted down (∼1 unit). Overall, despite some uncertainty in the electron density, we concluded that only the top 10% of the events are associated with strong scattering due to generated whistler‐mode waves, indicating strong scattering is not typically expected for these selected dispersionless injections.

## 5 Summary and Discussion

In this work, we investigated whistler‐mode wave activity in conjunction with energetic electron injections using in situ measurements by the THEMIS mission. This study relied on THEMIS‐D data from 2011 to 2020 when the spacecraft was in the magnetotail. We investigated the typical wave power immediately following injections. We also calculated the expected pitch angle diffusion rate resulting from the generated waves to investigate how frequently these waves cause strong scattering during injections. Finally, we studied the wave activity during DFBs as a subgroup of injections with a sharp rise in the northward magnetic field.

Our conclusions from this investigation are as follows:

- Superposed epoch analysis on the measured wave power of 733 selected dispersionless electron injection events indicates an enhancement in the wave power up to ∼2 times greater power at the onset in comparison with beforehand for most of the events.
- Only the top 5% of the selected events are associated with a significant enhancement in the wave power of around an order of magnitude.
- Waves generated during the injections present a heavy tail wave power distribution.
- Comparing calculated diffusion rates with strong diffusion shows that only the top 25% of the events are in the strong scattering limit, and only the top 10% show a measurable enhancement in the diffusion rate.
- Events with a sharp rise in the northward component of the magnetic field (DFBs) show at least two times greater associated wave power enhancement in the 95th percentile compared to other non‐DFB injections.

We observed that only the top 5% of the dispersionless injections are associated with whistler‐mode waves, not the majority of them. However, the reason is not evident in this study. Gabrielse et al. (2014) showed that only a subset of dispersionless injections as defined by their criteria had both electron and ion injection simultaneously. They observed many more singular injections (electron‐only or ion‐only injections) than coincident events, implying that the acceleration site is very narrow compared to the dawnward and duskward regions of the acceleration site where singular injections are observed. It suggests that some events selected were not at the very center of the acceleration region. Consequently, it might be possible that many of our selected events are also not located in the center of the acceleration region, which may explain the weak wave generation. However, in DFB injections we observed the same pattern, and again only the top 5% showed strong enhancement in wave power. Zhang et al. (2019) studied whistler‐mode waves surrounding dipolarization fronts and suggested the 10–20 keV electron populations are the major source of free energy that may exhibit perpendicular anisotropy and generate/amplify whistler‐mode waves. In comparison, the thermal electron population may show no anisotropy or parallel anisotropy. In this study, we have not classified different energy injections to investigate wave activity separately, but follow‐up research might consider classifying injections and elaborating on which group of injections lead to conditions to generate whistler‐mode waves more effectively.

Gabrielse et al. (2019) studied distinct types of injections that are observed by THEMIS. The typical large‐scale injections seen near 8 $R_{E}$ and/or at GEO are detectable in ground‐based observations. These usually expand over multiple MLT sectors and last tens of minutes. In contrast, small‐scale earthward‐propagating injections (minutes long or less and only a few $R_{E}$ expansion in *X* and *Y* directions) do not typically produce a notable precipitation signature in ground‐based riometer signals, or only result in a weak perturbation. They proposed these small‐scale injection regions may not contribute sufficient flux to cause strong scattering at the THEMIS location. In this study, we showed only 10% of the selected injections might cause strong pitch angle scattering due to the generated whistler‐mode waves. Lack of sufficient wave generation/amplification to interact with available particles and scatter them effectively might be a possible reason for the lack of relevant signature in the ground‐based observations for a group of injections. As future work, investigating the precipitation signature of the presented list of electron injections on ground instruments might clarify this speculation.

## Supporting information

Supporting Information S1

Data Set S1

## Data Availability

All the data for this study are available at the THEMIS data depository: http://themis.ssl.berkeley.edu/data/themis/. Data access and processing was done using SPEDAS software provided by the THEMIS mission (Angelopoulos et al., 2019).
