# Supplementary material for: Statistical Study of Whistler‐Mode Waves and Expected Pitch Angle Diffusion Rates During Dispersionless Electron Injections
Source: Geophys Res Lett. 2021 Sep 3;48(17):e2021GL094085. doi: 10.1029/2021GL094085 (PMC9286036; doi:10.1029/2021GL094085)
Supplement: Supplementary file 1 — Supporting Information S1 [file GRL-48-0-s001.pdf]

Supporting Information for "Statistical Study of Whistler-mode Waves and Expected Pitch Angle Diffusion Rates during Dispersionless Electron Injections"

R. Ghaffari[1], C. M. Cully[1], C. Gabrielse[2]

[1]Department of Physics and Astronomy, University of Calgary, Calgary, Alberta, Canada

[2]The Aerospace Corporation: El Segundo, California, US

Contents of this file

1. Figures S1 to S5

Additional Supporting Information (Files uploaded separately)

1. Caption for Data set S1

Introduction To have a better insight into the paper, we included the MLT distribution of the studied events in this supporting information file. We additionally classified the events into two groups based on their L value, $L > 10$ and $L < 10$, and regenerated figure 2 and figure 3 in the paper for these two groups.

Corresponding author: R. Ghaffari, Department of Physics and Astronomy, University of Calgary, Calgary, Alberta, Canada. (reihaneh.ghaffari@ucalgary.ca)

Figure S1. This figure demonstrates the studied injections' distribution in MLT domain. The events mostly occur far down tail. **Figure S2.** This figure is a superposed epoch analysis for the events with $L > 10$, similar to the figure 2 in the paper. Panel 'a' shows the integrated wave power for lower band chorus of all 627 events. Panels 'c' to 'e' are for 272, 209 and 146 events respectively that satisfy different DFB criteria as discussed in the paper. **Figure S3.** This figure is a superposed epoch analysis for the events with $L < 10$, similar to the figure 2 in the paper. Panel 'a' shows the integrated wave power for lower band chorus of all 106 events. Panels 'c' to 'e' are for 59, 48 and 34 events respectively that satisfy different DFB criteria as discussed in the paper. The wave power is more variant in this group (compared to figure S2), and as we could expect, it shows higher powers. **Figure S4.** Similar to figure 3 in the paper, this figure shows different percentiles of the calculated diffusion coefficient (panel a) and diffusion coefficients normalized to strong diffusion rate (panel b) for before and after the injection onset (solid and dashed curves respectively). Only the events with $L > 10$ have been considered in this figure. **Figure S5.** We only considered the events with $L < 10$ in this figure.

Data Set S1. List of the studied 733 selected dispersionless electron injection events.

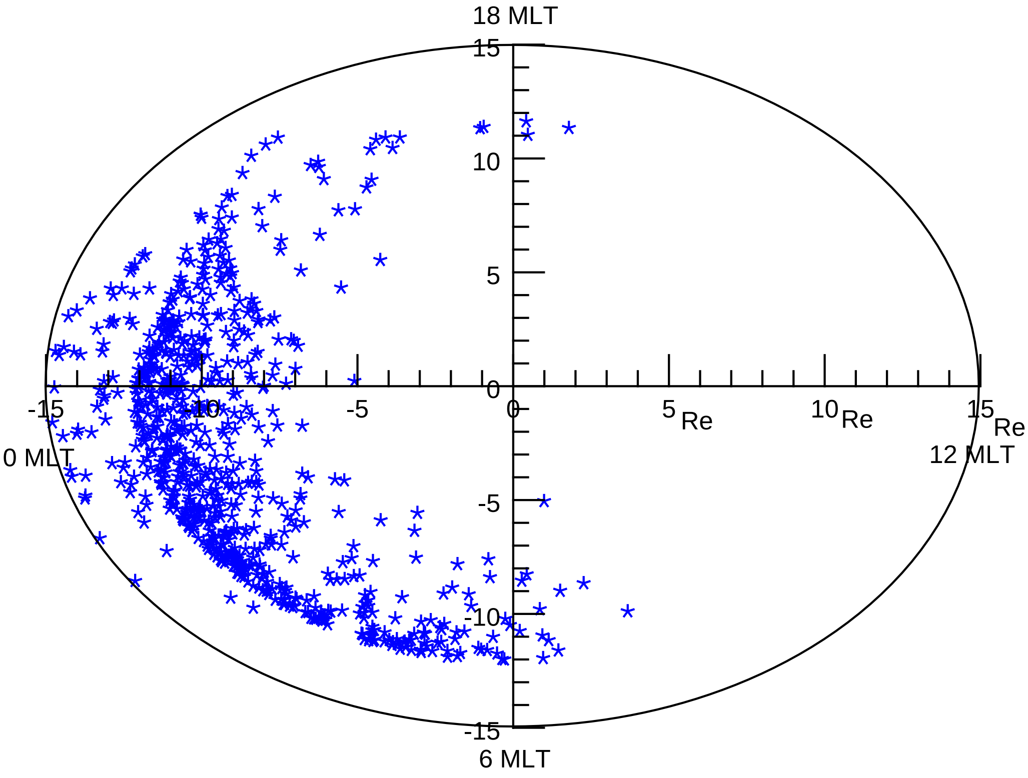

Figure S1. Injection events' distribution in L, MLT domain.

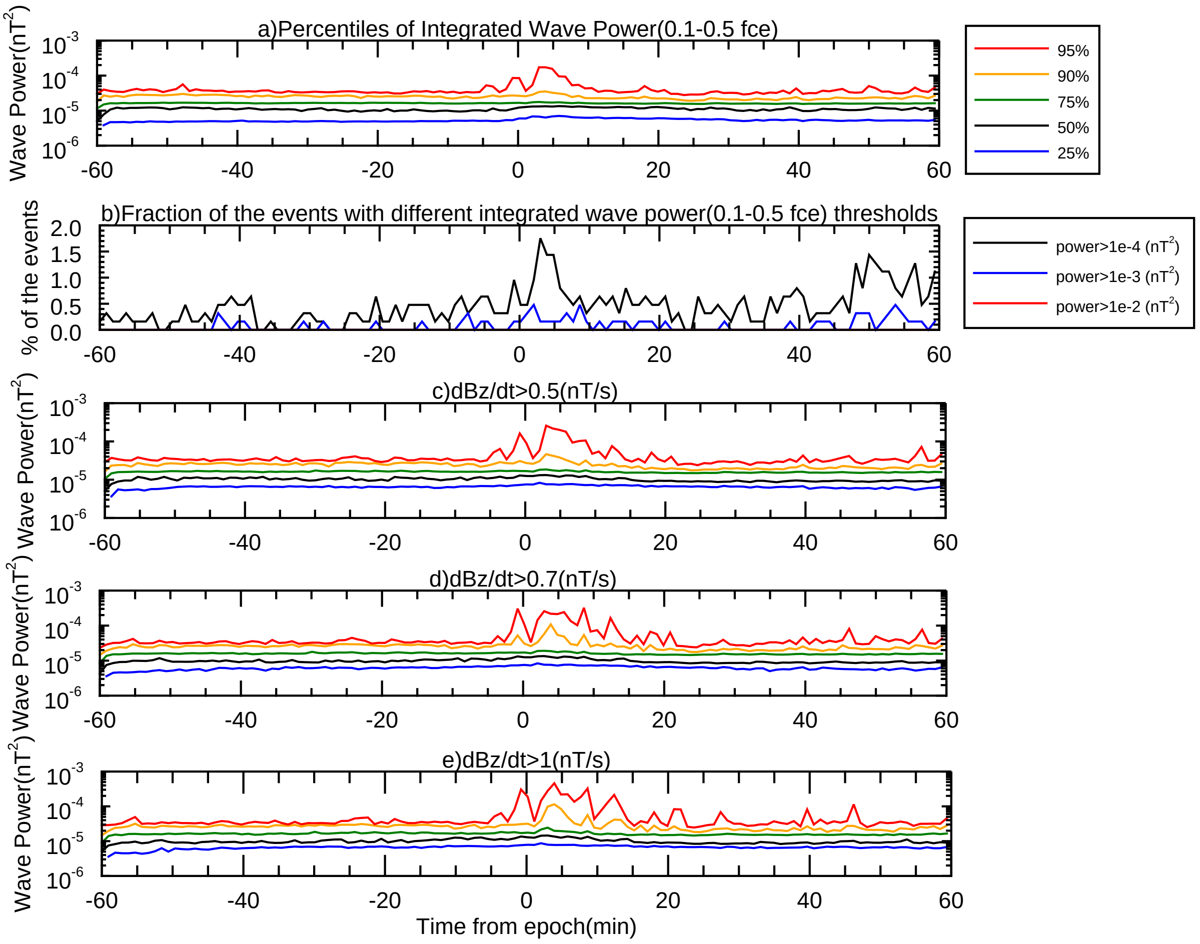

Figure S2. Same as figure 2 in the paper but for the events at $L > 10$

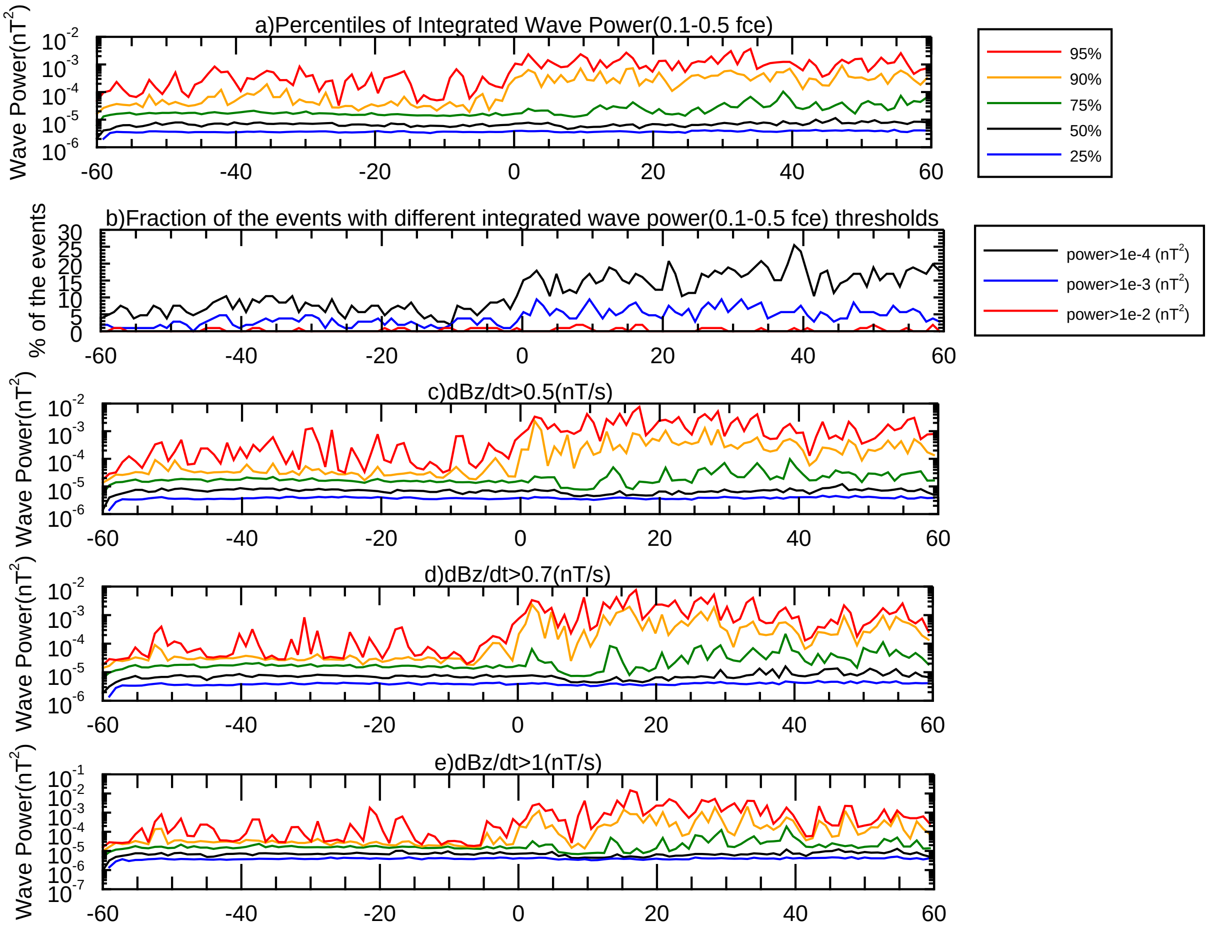

Figure S3. Same as figure 2 in the paper but for the events at $L < 10$

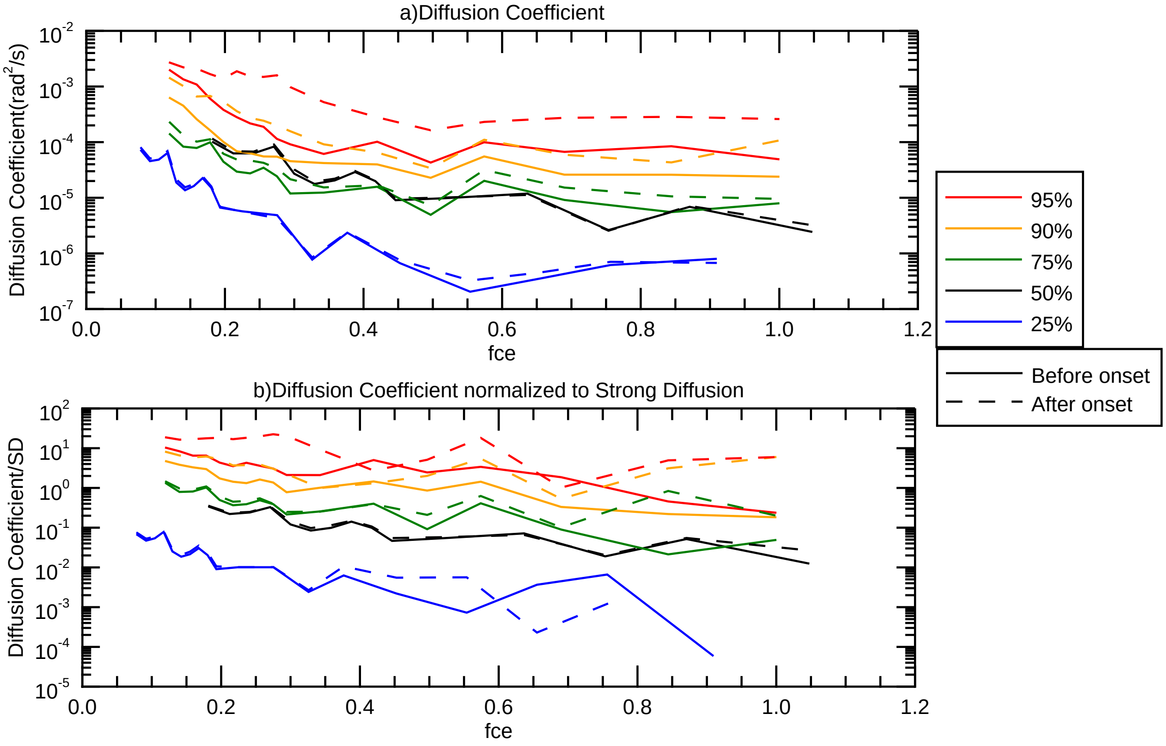

Figure S4. Same as figure 3 in the paper but for the events at $L > 10$

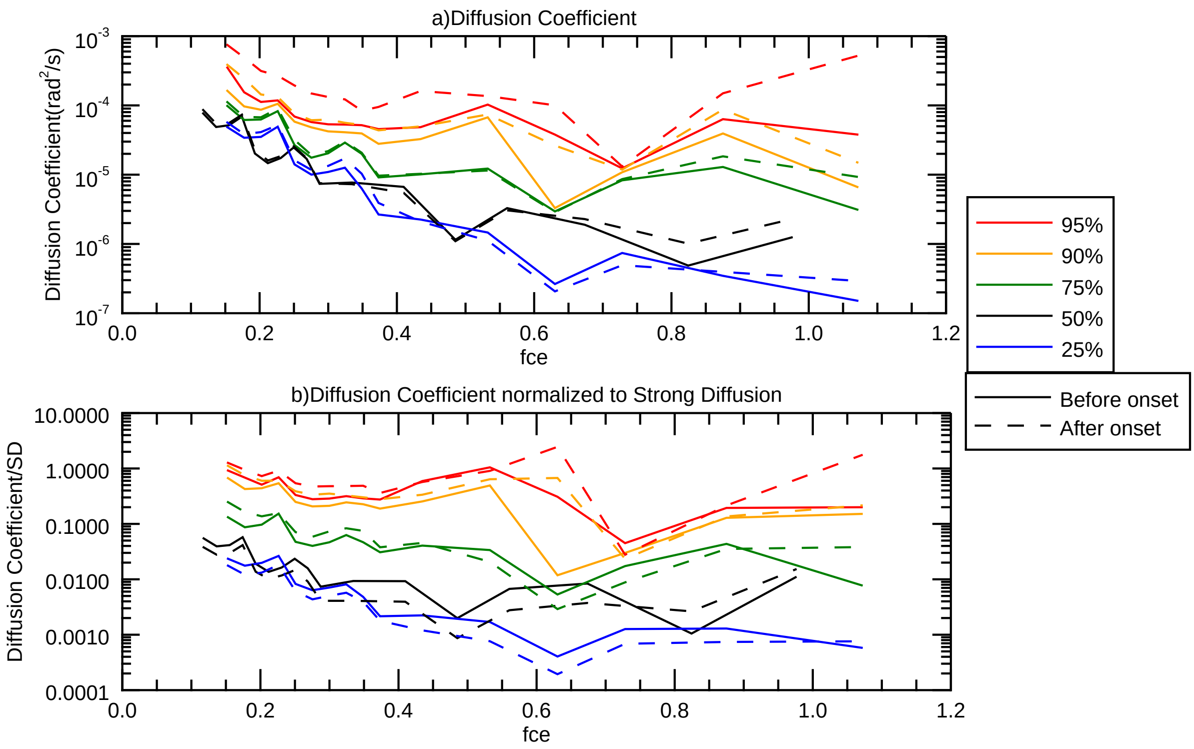

Figure S5. Same as figure 3 in the paper but for the events at $L < 10$
